# Supplementary material for: Validation of serum galactomannan antigen assay for invasive pulmonary aspergillosis mortality outcome prediction
Source: Microbiol Spectr. 2025 Oct 27;13(12):e00651-25. doi: 10.1128/spectrum.00651-25 (PMC12671216; doi:10.1128/spectrum.00651-25)
Supplement: Supplemental materials — Tables S1 to S5, Figures S1 to S4, and Supplemental information S1 and S2. [file spectrum.00651-25-s0001.docx]

**Supplementary Data**

**Appendix** (Sorted in order of appearance in article)

**Supplementary Table 1.** TRIPOD checklist: prediction model development

**Supplementary Figure 1.** Flowchart of inclusion and exclusion process

**Supplementary Figure 2.** PRISMA flowchart of systemic review and keyword search terms

**Supplementary Table 2.** Details of hematologic and non-hematologic malignancy, autoimmune disease, immunosuppressant, solid organ transplant

**Supplementary Table 3.** PRISMA checklist

**Supplementary Table 4.** Summary of systematic review

**Supplementary Table 5.** Univariate logistic regression analysis

**Supplementary Figure 3.** Receiver operating characteristic curve of the multivariable logistic regression significant sGMI markers

**Supplementary Figure 4.** Receiver operating characteristic curve of the multivariable logistic regression significant sGMI model performance

**Supplementary Information 1:** Logistic regression model development, specification details

**Table A.** Full multivariable logistic regression model with baseline sGMI ≥2 and day 7 sGMI ≥1.5 as marker

**Supplementary Information 2:** References of the Systematic Review of static and kinetic serum Galactomannan markers

**Supplementary Table 1.** TRIPOD checklist: prediction model development

| **Section/Topic** | **Item** | **Checklist Item** | **Manuscript Section** |
| --- | --- | --- | --- |
| **Title and abstract** | | | |
| Title | 1 | Identify the study as developing and/or validating a multivariable prediction model, the target population, and the outcome to be predicted. | Title, Abstract |
| Abstract | 2 | Provide a summary of objectives, study design, setting, participants, sample size, predictors, outcome, statistical analysis, results, and conclusions. | Abstract |
| **Introduction** | | | |
| Background and objectives | 3a | Explain the medical context (including whether diagnostic or prognostic) and rationale for developing or validating the multivariable prediction model, including references to existing models. | Introduction: section 1-3 |
|  | 3b | Specify the objectives, including whether the study describes the development or validation of the model or both. | Introduction: section 4 |
| **Methods** | | | |
| Source of data | 4a | Describe the study design or source of data (e.g., randomized trial, cohort, or registry data), separately for the development and validation data sets, if applicable. | Methods:  Patient population and data collection section 1 |
|  | 4b | Specify the key study dates, including start of accrual; end of accrual; and, if applicable, end of follow-up. | Methods:  Patient population and data collection section 1 |
| Participants | 5a | Specify key elements of the study setting (e.g., primary care, secondary care, general population) including number and location of centres. | Methods:  Patient population and data collection section 1 |
|  | 5b | Describe eligibility criteria for participants. | Methods:  Patient population and data collection section 1 |
|  | 5c | Give details of treatments received, if relevant. | Not relevant (validation study of prognostic markers) |
| Outcome | 6a | Clearly define the outcome that is predicted by the prediction model, including how and when assessed. | Methods:  Patient population and data collection section 2 |
|  | 6b | Report any actions to blind assessment of the outcome to be predicted. | Not applicable (Retrospective study) |
| Predictors | 7a | Clearly define all predictors used in developing or validating the multivariable prediction model, including how and when they were measured. | Methods: systematic review; Results: systematic review; Supplementary Data Table 4 |
|  | 7b | Report any actions to blind assessment of predictors for the outcome and other predictors. | Not applicable (Retrospective study) |
| Sample size | 8 | Explain how the study size was arrived at. | Method: Patient population and data collection; Statistical analysis: section 3; Supplementary information 1 |
| Missing data | 9 | Describe how missing data were handled (e.g., complete-case analysis, single imputation, multiple imputation) with details of any imputation method. | Method: Statistical analysis section 1 |
| Statistical analysis methods | 10a | Describe how predictors were handled in the analyses. | Method: Statistical analysis section 2 |
|  | 10b | Specify type of model, all model-building procedures (including any predictor selection), and method for internal validation. | Method: Statistical analysis section 2; Supplementary Information 1 |
|  | 10d | Specify all measures used to assess model performance and, if relevant, to compare multiple models. | Method: Statistical analysis section 2 |
| Risk groups | 11 | Provide details on how risk groups were created, if done. | Not applicable (validation study of prognostic markers) |
| **Results** | | | |
| Participants | 13a | Describe the flow of participants through the study, including the number of participants with and without the outcome and, if applicable, a summary of the follow-up time. A diagram may be helpful. | Results: cohort characteristics section 1; Supplementary Figure 2 |
|  | 13b | Describe the characteristics of the participants (basic demographics, clinical features, available predictors), including the number of participants with missing data for predictors and outcome. | Results: cohort characteristics section 2-3; Table 1; Supplementary Table 3 |
| Model development | 14a | Specify the number of participants and outcome events in each analysis. | Table 1; Supplementary Table 4 |
|  | 14b | If done, report the unadjusted association between each candidate predictor and outcome. | Supplementary Table 5 |
| Model specification | 15a | Present the full prediction model to allow predictions for individuals (i.e., all regression coefficients, and model intercept or baseline survival at a given time point). | Table 2a/2b; Table 3; Supplementary Information 2 |
|  | 15b | Explain how to the use the prediction model. | Table 2a/2b; Table 3 |
| Model performance | 16 | Report performance measures (with CIs) for the prediction model. | Results: Multivariable logistic regression analysis section 1-4 |
| **Discussion** | | | |
| Limitations | 18 | Discuss any limitations of the study (such as nonrepresentative sample, few events per predictor, missing data). | Discussion section 7 |
| Interpretation | 19b | Give an overall interpretation of the results, considering objectives, limitations, and results from similar studies, and other relevant evidence. | Discussion section 1-7 |
| Implications | 20 | Discuss the potential clinical use of the model and implications for future research. | Discussion section 2 |
| **Other information** | | | |
| Supplementary information | 21 | Provide information about the availability of supplementary resources, such as study protocol, Web calculator, and data sets. | Supplementary data |
| Funding | 22 | Give the source of funding and the role of the funders for the present study. | Transparency declaration: Funding |

**Supplementary Figure 1.** Flowchart of inclusion and exclusion process


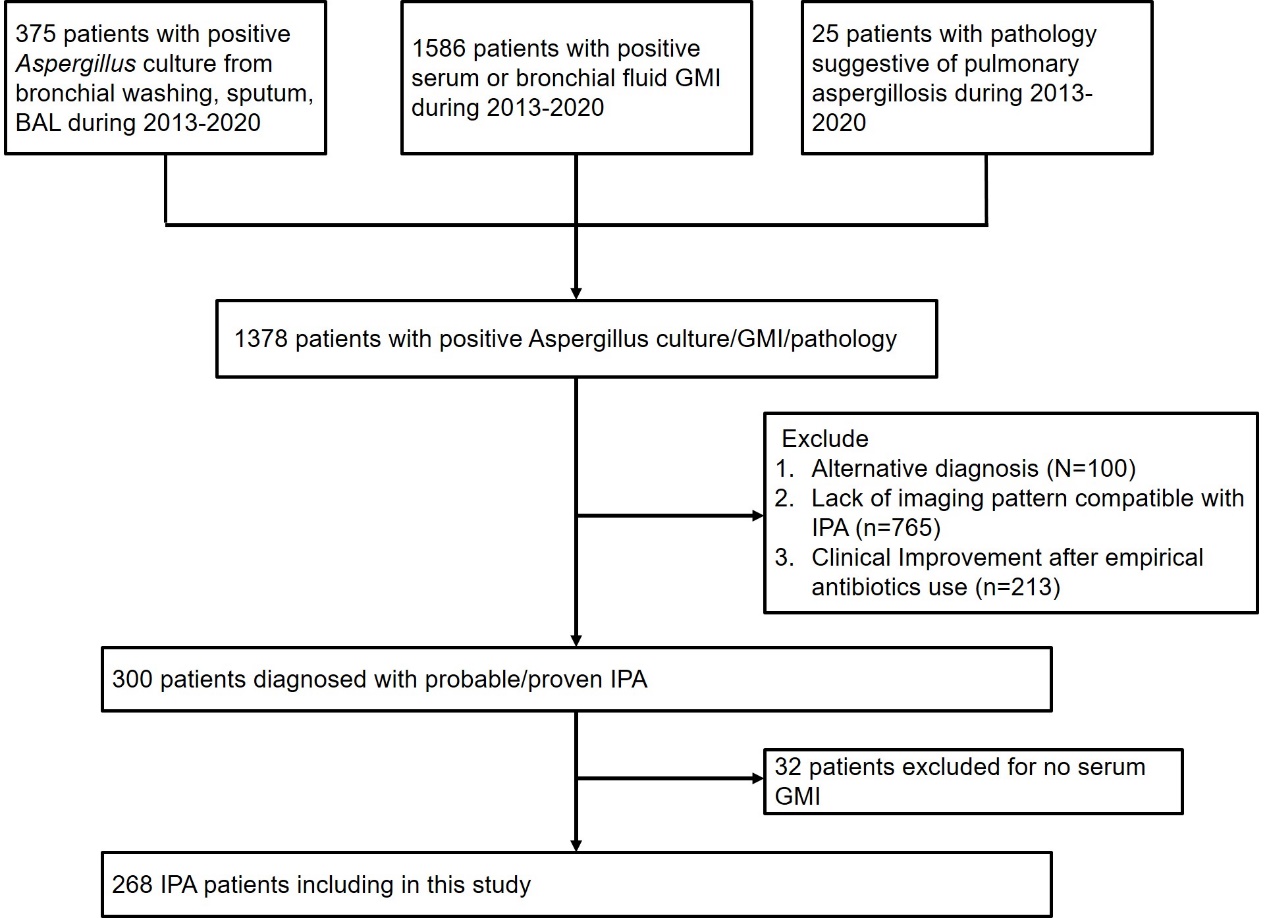


Abbreviations: BAL= bronchoalveolar lavage; GMI= galactomannan enzyme immunoassay optical density index; IPA= invasive pulmonary aspergillosis.

**Supplementary Figure 2.** PRISMA flowchart of systemic review and keyword search terms

Keyword search terms: ((Galactomannan [All Fields]) AND (Aspergillosis [All Fields]) AND (english[Language]) AND ((prognosis [All Fields]) OR (response [All Fields]) OR (therapy [All Fields]) OR (treatment [All Fields]) OR (therapeutics [All Fields]) OR (Risk [All Fields]) OR (outcome [All Fields])).

**Identification of studies via databases and registers**

Records identified from

Pubmed (n = 1021)

**Identification**

Reports excluded (n=993):

No evaluation of prognosis with galactomannan (n=388)

Review/Expert review/Editorial/Survey/To the editor (n=203)

Guidelines (n=12)

Meta-analysis (n=1)

Case report (n=126)

Did not involve humans (n=109)

Non-serum specimen (n=90)

Evaluation of false positivity of diagnosis (n=36)

Only chronic pulmonary aspergillosis or allergic bronchopulmonary aspergillosis included (n=15)

Included non-invasive aspergillus patient in analysis (n=5)

Non-aspergillosis related disease (n=3)

Findings not statistical significant (n=5)

Records screened

(n = 1021)

**Screening**

Reports sought for retrieval (n = 1021)

Reports assessed for eligibility

(n = 1021)

Studies included in review

(n = 28)

**Included**

**Supplementary Table 2.** Details of hematologic and non-hematologic malignancy, autoimmune disease, immunosuppressant, solid organ transplant.

| **Hematologic malignancy** | **N (%)** | **Non-hematologic malignancy** | **N (%)** | **Autoimmune disease** | **N (%)** | **Immuno-suppressant use** | **N (%)** | **Solid organ transplant** | **N (%)** |
| --- | --- | --- | --- | --- | --- | --- | --- | --- | --- |
| Acute myeloid leukaemia | 52 (32.7) | Lung | 13 (28.9) | Systemic lupus erythematosus | 10 (21.3) | Cyclosporine | 44 (47.8) | Lung | 3 (50.0) |
| Acute lymphocytic leukaemia | 26 (16.4) | Oropharyngeal | 8 (17.8) | Sjögren syndrome | 8 (17.0) | Tacrolimus | 26 (28.3) | Renal | 2 (33.3) |
| Myelodysplastic syndromes | 23 (14.5) | Breast | 5 (11.1) | Rheumatoid arthritis | 7 (14.9) | Azathioprine | 9 (9.8) | Liver | 1 (16.7) |
| Diffuse large B cell lymphoma | 17 (10.7) | Hepatocellular carcinoma | 3 (6.7) | Immune thrombocytopenia | 5 (10.6) | Mycophenolate | 7 (7.6) |  |  |
| Natural killer T cell Lymphoma | 5 (3.1) | Colon | 2 (4.4) | Antiphospholipid syndrome | 4 (8.5) | Leflunomide | 6 (6.5) |  |  |
| Chronic lymphocytic leukaemia/small lymphocytic lymphoma | 5 (3.1) | Cancer of unknown primary | 2 (4.4) | Autoimmune thyroiditis | 4 (8.5) | IVIG | 2 (2.2) |  |  |
| Hodgkin lymphoma | 4 (2.5) | Prostate | 2 (4.4) | Dermatomyositis/ Polymyositis | 3 (6.4) | Sirolimus | 1 (1.1) |  |  |
| Multiple myeloma | 5 (3.1) | Renal | 2 (4.4) | Autoimmune hemolytic anemia | 3 (6.4) | Abatacept | 1 (1.1) |  |  |
| Chronic myelogenous leukaemia | 4 (2.5) | Thymoma | 2 (4.4) | Microscopic Polyangiitis | 2 (4.3) | Cyclophosphamide | 1 (1.1) |  |  |
| Follicular lymphoma | 3 (1.9) | Thyroid | 2 (4.4) | Pemphigus/Pemphigoid | 1 (2.1) |  |  |  |  |
| Acute promyelocytic leukaemia | 3 (1.9) | Brain | 1 (2.2) | Seronegative Spondyloarthropathies | 1 (2.1) |  |  |  |  |
| Mantle cell lymphoma | 3 (1.9) | cervical | 1 (2.2) | Autoimmune hepatitis | 1 (2.1) |  |  |  |  |
| Burkitt lymphoma/leukaemia | 2 (1.3) | Esophageal | 1 (2.2) | Cryoglobulinemia | 1 (2.1) |  |  |  |  |
| Myeloid fibrosis | 2 (1.3) | Gastric | 1 (2.2) | Guillain-Barré Syndrome | 1 (2.1) |  |  |  |  |
| Mixed phenotype acute leukaemia | 1 (0.6) | Gastrointestinal stromal tumor | 1 (2.2) | Myasthenia gravis | 1 (2.1) |  |  |  |  |
| Lymphoblastic lymphoma/leukaemia | 1 (0.6) | Pancreas | 1 (2.2) | Systemic sclerosis | 1 (2.1) |  |  |  |  |
| MALToma | 1 (0.6) | Skin | 1 (2.2) | Undifferentiated connective tissue disease | 1 (2.1) |  |  |  |  |
| Chronic myelomonocytic leukaemia | 1 (0.6) |  |  |  |  |  |  |  |  |
| Low-grade B-cell lymphoma | 1 (0.6) |  |  |  |  |  |  |  |  |
| Myeloproliferative Neoplasm | 1 (0.6) |  |  |  |  |  |  |  |  |
| Peripheral T-cell lymphoma | 1 (0.6) |  |  |  |  |  |  |  |  |
| T-cell lymphoma | 1 (0.6) |  |  |  |  |  |  |  |  |
| Leukaemia | 1 (0.6) |  |  |  |  |  |  |  |  |
| Total | 159 |  | 45 |  | 47 |  | 92 |  | 6 |

**Supplementary Table 3.** PRISMA checklist

| **Section and Topic** | **Item #** | **Checklist item** | **Location where item is reported** |
| --- | --- | --- | --- |
| **TITLE** |  |  |  |
| Title | 1 | Identify the report as a systematic review. | None; The systematic review was performed to identify past serum galactomannan antigen EIA markers for our original study |
| **ABSTRACT** |  |  |  |
| Abstract | 2 | See the PRISMA 2020 for Abstracts checklist. | Abstract, not applicable for PRISMA 2020 abstract checklist due to the purpose of our study |
| **INTRODUCTION** |  |  |  |
| Rationale | 3 | Describe the rationale for the review in the context of existing knowledge. | Introduction: section 3-4 |
| Objectives | 4 | Provide an explicit statement of the objective(s) or question(s) the review addresses. | Methods: systematic review |
| **METHODS** |  |  |  |
| Eligibility criteria | 5 | Specify the inclusion and exclusion criteria for the review and how studies were grouped for the syntheses. | Methods: systematic review |
| Information sources | 6 | Specify all databases, registers, websites, organisations, reference lists and other sources searched or consulted to identify studies. Specify the date when each source was last searched or consulted. | Methods: systematic review; Supplementary Data Figure 1 |
| Search strategy | 7 | Present the full search strategies for all databases, registers and websites, including any filters and limits used. | Methods: systematic review; |
| Selection process | 8 | Specify the methods used to decide whether a study met the inclusion criteria of the review, including how many reviewers screened each record and each report retrieved, whether they worked independently, and if applicable, details of automation tools used in the process. | Methods: systematic review; Supplementary Data Figure 1 |
| Data collection process | 9 | Specify the methods used to collect data from reports, including how many reviewers collected data from each report, whether they worked independently, any processes for obtaining or confirming data from study investigators, and if applicable, details of automation tools used in the process. | Methods: systematic review |
| Data items | 10a | List and define all outcomes for which data were sought. Specify whether all results that were compatible with each outcome domain in each study were sought (e.g. for all measures, time points, analyses), and if not, the methods used to decide which results to collect. | Supplementary Data Table 4 |
|  | 10b | List and define all other variables for which data were sought (e.g. participant and intervention characteristics, funding sources). Describe any assumptions made about any missing or unclear information. | Supplementary Data Table 4 |
| Study risk of bias assessment | 11 | Specify the methods used to assess risk of bias in the included studies, including details of the tool(s) used, how many reviewers assessed each study and whether they worked independently, and if applicable, details of automation tools used in the process. | Methods: systematic review |
| Effect measures | 12 | Specify for each outcome the effect measure(s) (e.g. risk ratio, mean difference) used in the synthesis or presentation of results. | Supplementary Data Table 4 |
| Synthesis methods | 13a | Describe the processes used to decide which studies were eligible for each synthesis (e.g. tabulating the study intervention characteristics and comparing against the planned groups for each synthesis (item #5)). | Supplementary Figure 1 |
|  | 13b | Describe any methods required to prepare the data for presentation or synthesis, such as handling of missing summary statistics, or data conversions. | Supplementary Data Table 4 |
|  | 13c | Describe any methods used to tabulate or visually display results of individual studies and syntheses. | Supplementary Data Table 4 |
|  | 13d | Describe any methods used to synthesize results and provide a rationale for the choice(s). If meta-analysis was performed, describe the model(s), method(s) to identify the presence and extent of statistical heterogeneity, and software package(s) used. | Supplementary Data Table 4 |
|  | 13e | Describe any methods used to explore possible causes of heterogeneity among study results (e.g. subgroup analysis, meta-regression). | Supplementary Data Table 4 |
|  | 13f | Describe any sensitivity analyses conducted to assess robustness of the synthesized results. | No synthesis was done due to purpose of our study |
| Reporting bias assessment | 14 | Describe any methods used to assess risk of bias due to missing results in a synthesis (arising from reporting biases). | No synthesis was done due to purpose of our study |
| Certainty assessment | 15 | Describe any methods used to assess certainty (or confidence) in the body of evidence for an outcome. | Supplementary Data Table 4 |
| **RESULTS** |  |  |  |
| Study selection | 16a | Describe the results of the search and selection process, from the number of records identified in the search to the number of studies included in the review, ideally using a flow diagram. | Supplementary Data Figure 1 |
|  | 16b | Cite studies that might appear to meet the inclusion criteria, but which were excluded, and explain why they were excluded. | Supplementary Data Figure 1 |
| Study characteristics | 17 | Cite each included study and present its characteristics. | Supplementary Data Table 4 |
| Risk of bias in studies | 18 | Present assessments of risk of bias for each included study. | Study inclusion was performed irrespective of the risk of bias, since we would validate the findings with our cohort |
| Results of individual studies | 19 | For all outcomes, present, for each study: (a) summary statistics for each group (where appropriate) and (b) an effect estimate and its precision (e.g. confidence/credible interval), ideally using structured tables or plots. | Supplementary Data Table 4 |
| Results of syntheses | 20a | For each synthesis, briefly summarise the characteristics and risk of bias among contributing studies. | Supplementary Data Table 4 |
|  | 20b | Present results of all statistical syntheses conducted. If meta-analysis was done, present for each the summary estimate and its precision (e.g. confidence/credible interval) and measures of statistical heterogeneity. If comparing groups, describe the direction of the effect. | Supplementary Data Table 4 |
|  | 20c | Present results of all investigations of possible causes of heterogeneity among study results. | No synthesis was done due to the nature of this study was served only to identify markers for validation but not generating results. |
|  | 20d | Present results of all sensitivity analyses conducted to assess the robustness of the synthesized results. | No synthesis was done due to the nature of this study was served only to identify markers for validation but not generating results. |
| Reporting biases | 21 | Present assessments of risk of bias due to missing results (arising from reporting biases) for each synthesis assessed. | Study inclusion was performed irrespective of the risk of bias, since we would validate the findings with our cohort |
| Certainty of evidence | 22 | Present assessments of certainty (or confidence) in the body of evidence for each outcome assessed. | Supplementary Data Table 4 |
| **DISCUSSION** |  |  |  |
| Discussion | 23a | Provide a general interpretation of the results in the context of other evidence. | Result: systematic review; Supplementary Data Table 4 |
|  | 23b | Discuss any limitations of the evidence included in the review. | Discussion: section 1-6 |
|  | 23c | Discuss any limitations of the review processes used. | Discussion: section 1-6 |
|  | 23d | Discuss implications of the results for practice, policy, and future research. | Conclusion |
| **OTHER INFORMATION** |  |  |  |
| Registration and protocol | 24a | Provide registration information for the review, including register name and registration number, or state that the review was not registered. | This review was not registered |
|  | 24b | Indicate where the review protocol can be accessed, or state that a protocol was not prepared. | Methods: systematic review; Supplementary Data Figure 1 |
|  | 24c | Describe and explain any amendments to information provided at registration or in the protocol. | Not applicable |
| Support | 25 | Describe sources of financial or non-financial support for the review, and the role of the funders or sponsors in the review. | No financial support was received. |
| Competing interests | 26 | Declare any competing interests of review authors. | All authors report no conflicts of interest relevant to this article |
| Availability of data, code and other materials | 27 | Report which of the following are publicly available and where they can be found: template data collection forms; data extracted from included studies; data used for all analyses; analytic code; any other materials used in the review. | Supplementary Data Figure 1, Supplementary Data Table 4, Supplementary Data Information 2 |

*From:*  Page MJ, McKenzie JE, Bossuyt PM, Boutron I, Hoffmann TC, Mulrow CD, et al. The PRISMA 2020 statement: an updated guideline for reporting systematic reviews. BMJ 2021;372:n71. doi: 10.1136/bmj.n71. This work is licensed under CC BY 4.0. To view a copy of this license, visit <https://creativecommons.org/licenses/by/4.0/>

**Supplementary Table 4.** Summary of systematic review

| **Required parameter** | **Parameter detail** | **Study population** | **IPA definition** | **Sample size** | **Predicted outcome and result** | **95% CI** | ***p*-value** | **References** |  |  |  |
| --- | --- | --- | --- | --- | --- | --- | --- | --- | --- | --- | --- |
| Baseline sGMI markers | |  |  |  | |  | | |  |  |  |
| Baseline sGMI | Baseline sGMI | Kidney transplant recipients | EORTC/MSG 2008; Proven/Probable | 41 | 12-week mortality: unadjusted HR 1.371 per unit GMI increase | 1.12-1.67 | 0.002 | Heylen et al. (1) |  |  |  |
|  | Baseline sGMI | Malignancy, SOT, and others | EORTC/MSG 2008; Proven/Probable | 93 | 6-week mortality: adjusted HR 1.25 per unit GMI increase | 1.01-1.54 | 0.039 | Koo et al. (2) |  |  |  |
|  | Baseline sGMI | Malignancy, SOT, and others | EORTC/MSG 2008; Proven/Probable | 93 | 12-week mortality: adjusted HR 1.27 per unit GMI increase | 1.03-1.55 | 0.024 | Koo et al. (2) |  |  |  |
|  | Baseline sGMI | Hematological malignancy and others | EORTC/MSG 2008; Proven/Probable/Possible | 57 | 60-day mortality: HR 1.25 per unit GMI increase | 1.10-1.43 | <0.05 | Bergeron et al. (3) |  |  |  |
|  | Baseline sGMI | Hematological malignancy, post-HSCT, and others | EORTC/MSG 2008; Proven/Probable; GCA and the CAS cohort | 251 | 6-week response: adjusted OR 0.97 per 0.1 unit GMI increase | 0.95-0.99 | <0.01 | Pang et al. (4) |  |  |  |
|  | Baseline sGMI | Hematological malignancy, post-HSCT, and others | EORTC/MSG 2008; Proven/Probable; GCA and the CAS cohort | 251 | 12-week survival: adjusted OR 0.97 per 0.1 unit GMI increase | 0.96-0.99 | <0.01 | Pang et al. (4) |  |  |  |
| Baseline sGMI with cutoff | |  |  |  | |  | | |  |  |  |
| 0.5 | Baseline sGMI < 0.5 | Hematological malignancy with prolong neutropenia | EORTC/MSG 2008; Proven/Probable | 30 | Week 4 treatment response with sGMI < 0.5 and ≥0.5: 100% and 54% | N/A | 0.005 | Couchepin et al. (5) |  |  |  |
|  | Baseline sGMI < 0.5 | Hematological malignancy with prolong neutropenia | EORTC/MSG 2008; Proven/Probable | 30 | 12-week survival with sGMI < 0.5 and ≥0.5: 100% and 57% | N/A | 0.006 | Couchepin et al. (5) |  |  |  |
|  | Baseline sGMI < 0.5 | Malignancy, SOT, post-HSCT, and others | EORTC/MSG 2008; Proven/Probable/Possible | 47 | 6-week treatment response: unadjusted OR 4.5 | 1.09-23.52 | 0.05 | Neofytos et al. (6) |  |  |  |
|  | Baseline sGMI < 0.5 | Malignancy, SOT, post-HSCT, and others | EORTC/MSG 2008; Proven/Probable/Possible | 47 | 12-week survival: unadjusted OR 7.0 | 1.42-38.74 | 0.02 | Neofytos et al. (6) |  |  |  |
|  | Baseline sGMI < 0.5 | SOT | EORTC/MSG 2008; Proven/Probable | 24 | 30-day survivors and non-survivors: 56% and 18% | N/A | 0.021 | Hoyo et al. (7) |  |  |  |
|  | Baseline sGMI ≥ 0.5 | Hematological malignancy, post-HSCT, SOT, and others | EORTC/MSGERC 2019; Proven/Probable/Possible | 40 | 6-week survival with sGMI < 0.5 and ≥0.5: 84% and 53.3% | N/A | 0.04 | Moreno et al. (8) |  |  |  |
|  | Baseline sGMI ≥ 0.5 | Allogeneic HSCT recipient | EORTC/MSG 2008; Proven/Probable | 100 | 6-week respiratory mortality: adjusted HR 3.01 | 1.06-8.53 | 0.038 | Fisher et al. (9) |  |  |  |
|  | Baseline sGMI ≥ 0.5 | Hematological malignancy | EORTC/MSG 2008; Probable/Possible | 391 | Poor outcome (self-defined): adjusted HR 2.28 | 1.10-4.69 | 0.026 | Kim et al. (10) |  |  |  |
| 1 | Baseline sGMI ≥ 1.0 | Allogeneic HSCT recipient | EORTC/MSG 2008; Proven/Probable | 100 | 180-day respiratory mortality: adjusted HR 2.54 | 1.13-5.69 | 0.024 | Fisher et al. (9) |  |  |  |
|  | Baseline sGMI ≥ 1.0 | Allogeneic HSCT recipient | EORTC/MSG 2008; Proven/Probable | 100 | 180-day survival: adjusted HR 2.12 | 1.10-4.06 | 0.024 | Fisher et al. (9) |  |  |  |
|  | Baseline sGMI ≥ 1.0 | Allogeneic HSCT recipient | EORTC/MSG 2008; Proven/Probable | 100 | 6-week respiratory mortality: adjusted HR 4.09 | 1.33-12.5 | 0.014 | Fisher et al. (9) |  |  |  |
|  | Baseline sGMI ≥ 1.0 | Hematological malignancy, HSCT recipient, SOT, and others | EORTC/MSGERC 2019; Proven/Probable/Possible | 40 | 6-week survival with sGMI < 0.5 and ≥0.5: 82.8% and 45.5% | N/A | 0.02 | Moreno et al. (8) |  |  |  |
| 1.5 | Baseline sGMI ≥ 1,5 | Allogeneic HSCT recipient | EORTC/MSG 2008; Proven/Probable | 100 | 180-day survival: adjusted HR 2.95 | 1.47-5.91 | 0.002 | Fisher et al. (9) |  |  |  |
|  | Baseline sGMI ≥ 1.5 | Allogeneic HSCT recipient | EORTC/MSG 2008; Proven/Probable | 100 | 180-day respiratory mortality: adjusted HR 2.96 | 1.26-6.98 | 0.013 | Fisher et al. (9) |  |  |  |
|  | Baseline sGMI ≥ 1.5 | Allogeneic HSCT recipient | EORTC/MSG 2008; Proven/Probable | 100 | 6-week respiratory mortality: adjusted HR 5.09 | 1.64-15.8 | 0.005 | Fisher et al. (9) |  |  |  |
| 2 | Baseline sGMI ≥ 2.0 | Allogeneic HSCT recipient | EORTC/MSG 2008; Proven/Probable | 100 | 6-week respiratory mortality: adjusted HR 6.56 | 1.88-22.9 | 0.003 | Fisher et al. (9) |  |  |  |
|  | Baseline sGMI ≥ 2.0 | Allogeneic HSCT recipient | EORTC/MSG 2008; Proven/Probable | 100 | 180-day respiratory mortality: adjusted HR 4.01 | 1.58-10.1 | 0.003 | Fisher et al. (9) |  |  |  |
|  | Baseline sGMI ≥ 2.0 | Allogeneic HSCT recipient | EORTC/MSG 2008; Proven/Probable | 100 | 180-day survival: adjusted HR 4.08 | 1.68-9.87 | 0.002 | Fisher et al. (9) |  |  |  |
| Baseline mean/median sGMI | Baseline median sGMI | Pediatric hematological malignancy and SAA | EORTC/MSG 2008; Proven/Probable | 45 | 12-week survivors and non-survivors: 0.46 and 1.21 | N/A | 0.015 | Han et al. (11) |  |  |  |
|  | Baseline median sGMI | Hematological malignancy and others | EORTC/MSG 2008; Proven/Probable | 40 | 12-week survivors and non-survivors: 0.9 and 4.3 | N/A | 0.047 | Vehreschild et al. (12) |  |  |  |
|  | Baseline mean sGMI | Kidney transplant recipients | EORTC/MSG 2008; Proven/Probable | 112 | 6-week survivors and non-survivors: 0.5 and 1.1 | N/A | 0.024 | López -Medrano et al. (13) |  |  |  |
| Other sGMI markers | |  |  |  |  |  |  |  |  |  |  |
| sGMI at day 7 | Week 1 median sGMI | Pediatric hematological malignancy and SAA | EORTC/MSG 2008; Proven/Probable | 45 | 12-week survivors and non-survivors: 0.39 and 1.64 | N/A | 0.001 | Han et al. (11) |  |  |  |
|  | Week 1 sGMI > 1.5 | Pediatric hematological malignancy and SAA | EORTC/MSG 2008; Proven/Probable | 45 | 12-week mortality prediction: sensitivity 61.5%, specificity 89.3%, negative predictive value 83.3%, positive predictive value 72.7% | N/A | N/A | Han et al. (11) |  |  |  |
|  | Mean sGMI at day 7 | Hematological malignancy, post-HSCT and others; baseline sGMI>0.5 | EORTC/MSG 2008; Proven/Probable/Possible; GCA cohort | 71 | 12-week responders and non-responders: week 1 GMIs of 0.62 ± 0.12 and 1.15 ± 0.22 | N/A | 0.035 | Chai et al. (14) |  |  |  |
|  | sGMI < 0.5 within 7 days | Multiple myeloma with neutropenia | EORTC/MSG 2008; Proven/Probable/Probable without prespecified radiologic findings | 98 | 6-week success treatment response: adjusted OR 2.9 | 1.01- 8.33 | 0.048 | Nouér et al. (15) |  |  |  |
|  | sGMI < 0.5 within 7 days | Multiple myeloma with neutropenia | EORTC/MSG 2008; Proven/Probable/Probable without prespecified radiologic findings | 98 | 6-week survivors and non-survivors: 45.5% and 22.6% | N/A | 0.03 | Nouér et al. (15) |  |  |  |
|  | Week 1 sGMI – baseline sGMI | Hematological malignancy, post-HSCT, and others | EORTC/MSG 2008; Proven/Probable; GCA and the CAS cohort | 251 | 6-week success treatment response: adjusted OR 0.98 per 0.1 sGMI increase | 0.96-1.00 | 0.03 | Pang et al. (4) |  |  |  |
|  | Week 1 sGMI – baseline sGMI | Hematological malignancy, post-HSCT, and others | EORTC/MSG 2008; Proven/Probable; GCA and the CAS cohort | 251 | 12-week survival: adjusted OR 0.97 per 0.1 sGMI increase | 0.96-0.99 | <0.01 | Pang et al. (4) |  |  |  |
|  | Week 1 sGMI – baseline sGMI | Hematological malignancy, post HSCT, and others | EORTC/MSG 2008; Proven/Probable; GCA cohort | 70 | 12-week responders in voriconazole treated group had greater decline | N/A | 0.001 | Chai et al. (16) |  |  |  |
|  | (Baseline sGMI – week 1 sGMI)/ days between tests | Malignancy, SOT, and others | EORTC/MSG 2008; Proven/Probable | 93 | 6-week survival: adjusted HR 0.78 | 0.63-0.96 | 0.02 | Koo et al. (2) |  |  |  |
|  | (Baseline sGMI – week 1 sGMI)/ days between tests | Malignancy, SOT, and others | EORTC/MSG 2008; Proven/Probable | 93 | 6-week survival: adjusted HR 0.75 | 0.61-0.92 | 0.006 | Koo et al. (2) |  |  |  |
| sGMI at day 14 | Week 2 median sGMI | Pediatric hematological malignancy and SAA | EORTC/MSG 2008; Proven/Probable | 45 | 12-week survivors and non-survivors: 0.38 and 2.76 | N/A | 0.004 | Han et al. (11) |  |  |  |
|  | Mean sGMI at day 14 | Malignancy, SOT, post-HSCT, and others | EORTC/MSG 2008; Proven/Probable/Possible | 47 | Mean difference 0.72 between 12-week responders and non-responders | 0.13-1.29 | 0.02 | Neofytos et al. (6) |  |  |  |
|  | Week 2 sGMI – baseline sGMI | Hematological malignancy, post-HSCT and others | EORTC/MSG 2008; GCA and the CAS cohort; Proven/Probable | 251 | 6-week success treatment response: adjusted OR 0.97 per 0.1 sGMI increase | 0.94-1.00 | 0.03 | Pang et al. (4) |  |  |  |
|  | Week 2 sGMI – baseline sGMI | Hematological malignancy, post-HSCT and others | EORTC/MSG 2008; GCA and the CAS cohort; Proven/Probable | 251 | 12-week survival: adjusted OR 0.96 per 0.1 sGMI increase | 0.94-0.99 | <0.01 | Pang et al. (4) |  |  |  |
|  | Week 2 sGMI – baseline sGMI | Hematological malignancy, post-HSCT, and others | EORTC/MSG 2008; Proven/Probable/Possible; GCA cohort | 131 | 12-week non-responders with increased week 2 sGMI – baseline sGMI change | N/A | 0.001 | Chai et al. (14) |  |  |  |
|  | Week 2 sGMI – baseline sGMI | Hematological malignancy, post-HSCT, and others | EORTC/MSG 2008; Proven/Probable/Possible; GCA cohort | 131 | 12-week non-survivors with increased week 2 sGMI – baseline sGMI change | N/A | <0.001 | Chai et al. (14) |  |  |  |
|  | Week 2 sGMI – baseline sGMI | Hematological malignancy, post-HSCT, and others | EORTC/MSG 2008; Proven/Probable/Possible; GCA cohort | 131 | 12-week unsatisfactory treatment response likelihood increased by 21.6% per unit sGMI increase | 19.4-23.8 | 0.018 | Chai et al. (14) |  |  |  |
|  | Week 2 sGMI – baseline sGMI | Hematological malignancy, post-HSCT, and others | EORTC/MSG 2008; Proven/Probable; GCA cohort | 70 | 12-week responders in voriconazole treated group had greater decline | N/A | 0.046 | Chai et al. (16) |  |  |  |
|  | Week 2 sGMI – Week 1 sGMI | Hematological malignancy, post-HSCT, and others | EORTC/MSG 2008; Proven/Probable/Possible; GCA cohort | 131 | 12-week non-responders with increased week 2 sGMI – week 1 sGMI change | N/A | 0.022 | Chai et al. (14) |  |  |  |
|  | Week 2 sGMI – Week 1 sGMI | Hematological malignancy, post-HSCT, and others | EORTC/MSG 2008; Proven/Probable/Possible; GCA cohort | 131 | 12-week non-survivors with increased week 2 sGMI – week 1 sGMI change | N/A | <0.001 | Chai et al. (14) |  |  |  |
|  | Baseline sGMI – Week 2 sGMI | Malignancy, SOT, post-HSCT, and others | EORTC/MSG 2008; Proven/Probable/Possible | 47 | Mean difference 0.58 between 6-week responders and non-responders | 0.09-1.07 | 0.03 | Neofytos et al. (6) |  |  |  |
| sGMI over 1 month | |  |  |  |  |  |  |  |  |  |  |
| 6 week | Baseline sGMI – 6-week sGMI | Malignancy, SOT, post-HSCT, and others | EORTC/MSG 2008; Proven/Probable/Possible | 47 | Mean difference 0.65 between 6-week responders and non-responders | 0.06-1.23 | 0.03 | Neofytos et al. (6) |  |  |  |
|  | Baseline sGMI – 6-week sGMI | Malignancy, SOT, post-HSCT, and others | EORTC/MSG 2008; Proven/Probable/Possible | 47 | Mean difference 0.98 between 12-week responders and non-responders | 0.30-1.66 | 0.01 | Neofytos et al. (6) |  |  |  |
| 3 month | sGMI persistently > 0.5 for within 3 months | Hematological malignancy, SOT, post-HSCT, SAA | EORTC/MSG 2008; Proven/Probable | 110 | 90-day mortality: adjusted HR 7.14 | 3.33-14.28 | <0.001 | Park S.Y. et al. (17) |  |  |  |
| sGMI during treatment | |  |  |  | |  | | |  |  |  |
| sGMI< 0.5 for 2 weeks | sGMI persistently < 0.5 for 2 weeks | Multiple myeloma (91%) and others | EORTC 2002; Proven/Probable | 56 | Excellent correlation with mortality (**Κ**= 0.861) | 0.71-1.00 | <0.001 | Woods et al. (18) |  |  |  |
|  | sGMI persistently < 0.5 for 2 weeks | Multiple myeloma (91%) and others | EORTC 2002; Proven/Probable | 56 | Lower mortality | N/A | <0.001 | Woods et al. (18) |  |  |  |
|  | sGMI persistently < 0.5 for 2 weeks | Hematological malignancy | EORTC 2002; Proven/Probable | 70 | Good correlation with 6-week mortality (**Κ**= 0.588) | 0.40-0.77 | <0.05 | Maertens et al. (19) |  |  |  |
|  | sGMI persistently < 0.5 for 2 weeks | Hematological malignancy | EORTC 2002; Proven/Probable | 70 | Excellent correlation with 12-week mortality (**Κ**= 0.886) | 0.78-0.995 | <0.05 | Maertens et al. (19) |  |  |  |
|  | sGMI persistently < 0.5 for 2 weeks | Hematological malignancy | EORTC 2002; Proven/Probable | 70 | Excellent correlation with 6-week treatment response (**Κ**= 0.752) | 0.58-0.92 | <0.05 | Maertens et al. (19) |  |  |  |
|  | sGMI persistently < 0.5 for 2 weeks | Hematological malignancy | EORTC 2002; Proven/Probable | 70 | Lower mortality | N/A | <0.001 | Maertens et al. (19) |  |  |  |
|  | sGMI persistently < 0.5 for 2 weeks | Multiple myeloma (90%) and others | EORTC/MSG 2008; Proven/Probable | 115 | Excellent correlation with 6-week treatment response (**Κ**= 0.819) | N/A | <0.001 | Nouér et al. (20) |  |  |  |
|  | sGMI persistently < 0.5 for 2 weeks | Multiple myeloma (90%) and others | EORTC/MSG 2008; Proven/Probable | 115 | 6-week treatment response prediction: sensitivity 100%, specificity 76% | N/A | N/A | Nouér et al. (20) |  |  |  |
|  | sGMI persistently < 0.5 for 2 weeks | Acute leukaemia | EORTC/MSG 2008; Proven/Probable | 58 | Good correlation with 6-week clinical failure (**Κ**= 0.663) | 0.47-0.86 | <0.05 | Park S.H. et al. (21) |  |  |  |
|  | sGMI persistently < 0.5 for 2 weeks | Acute leukaemia | EORTC/MSG 2008; Proven/Probable | 58 | Excellent correlation with 12-week clinical failure (**Κ**= 0.819) | 0.67-0.91 | <0.05 | Park S.H. et al. (21) |  |  |  |
|  | sGMI persistently < 0.5 for 2 weeks | Malignancy, SOT, post-HSCT, and others | EORTC/MSG 2008; Proven/Probable/Possible | 47 | 12-week success treatment response: OR 4.5 | 1.0-27.0 | 0.05 | Neofytos et al. (6) |  |  |  |
|  | sGMI persistently < 0.5 for 2 weeks | Malignancy, SOT, post-HSCT, and others | EORTC/MSG 2008; Proven/Probable/Possible | 47 | 12-week survival: OR 6.5 | 1.3-33.0 | 0.02 | Neofytos et al. (6) |  |  |  |
| sGMI persistently > 1.0 | sGMI persistently > 1.0 | Hematological malignancy, post-HSCT | Self-defined diagnosis/ Proven/Probable | 18 | Survivors and non-survivors: 100% and 20% had sGMI persistently > 1.0 | N/A | 0.002 | Salonen et al. (22) |  |  |  |
| Maximum sGMI | Maximum sGMI | Hematological malignancy, SOT, and others | EORTC/MSGERC 2019 and AspICU; Proven/Probable/Possible/Putative | 125 | 3-year mortality: adjusted HR 1.15 | 1.04-1.27 | 0.005 | Chen et al. (23) |  |  |  |
|  | Maximum sGMI – baseline sGMI | Mixed ICU | EORTC/MSG 2008; Proven/Probable/Possible | 160 | Significantly higher in ICU non-survivors (Only shown in box plot) | N/A | 0.027 | Teering et al. (24) |  |  |  |
| sGMI trajectory | Increasing sGMI | Malignancy, SOT, post-HSCT, and others | EORTC/MSG 2008; Proven/Probable/Possible/no IA | 57 | 30-day survivors and non-survivors: 5.4% and 64.9% | N/A | 0.02 | Khanna et al. (25) |  |  |  |
|  | Increasing sGMI | Medical ICU | Self-defined diagnosis | 235 | 30-day mortality: OR 6.37 | 3.06-13.24 | <0.001 | Dabas et al. (26) |  |  |  |
|  | High risk: Baseline sGMI >1.4+ Week 1 sGMI >0.4 | Hematology | EORTC/MSG 2008; Proven/Probable | 285 | 6-week mortality in high risk group and intermediate+ low risk group: 40.6% and 17.1% | N/A | <0.001 | Mercier et al. (27) |  |  |  |
|  | Baseline <0.5 with rise to >0.5 at week 2 | Hematological malignancy, post-HSCT, and others | EORTC/MSG 2008; Proven/Probable/Possible; GCA cohort | 131 | 12-week non-survivor: likelihood ratio 4.48 | 2.3-8.71 | <0.001 | Chai et al. (14) |  |  |  |
|  | Doubling in sGMI | Critical ill COVID-19 in ICU | ECMM/ISHAM 2021; Probable/Possible | 207 | In-hospital mortality: adjusted HR 3.07 | 1.14-12.91 | 0.01 | Er et al. (28) |  |  |  |

Of the 26 different sGMI markers, static markers using the mean, median, or maximum sGMI were discarded because their value differed between studies and were unlikely to extrapolate to clinical use (n=7). The baseline sGMI cutoff of 0.5 was not applied because it is the proposed cutoff level for ICU patients in our study (n=1). The kinetic markers involving a cutoff of 0.5 or less (n=4) and markers with sGMI follow-up over 1 month (n=2) were not investigated as all the follow-up sGMIs were above 0.5 with limited follow-up data among 1 month.

Abbreviations: 95% CI= 95% confidence interval; sGMI= serum galactomannan enzyme immunoassay optical density index; GMI= galactomannan enzyme immunoassay optical density index; HR= Hazard ratio; EORTC/MSG= European Organization for Research and Treatment of Cancer and the Mycoses Study Group; SOT=Solid organ transplant; HSCT= hematopoietic stem cell transplantation; OR= Odds ratio; GCA= Global Comparative Aspergillosis Study; CAS= Combination Antifungal Study; N/A= Not available in study; SAA= severe aplastic anemia; **κ**= kappa coefficient; ICU=Intensive care unit; ECMM/ISHAM= European Confederation of Medical Mycology and the International Society for Human and Animal Mycology

1. Malignancy applies to hematological and non-hematological malignancies
2. References listed in Supplementary Data due to limitation of reference numbers

**Supplementary Table 5.** Univariate logistic regression analysis

|  | 30-day mortality | | 90-day mortality | | In-hospital mortality | |
| --- | --- | --- | --- | --- | --- | --- |
|  | Univariate OR (95% CI) | *p* value | Univariate OR (95% CI) | *p* value | Univariate OR (95% CI) | *p* value |
| Age, years old | 1.01 (1.00-1.03) | **0.046** | 1.01 (1.00-1.02) | 0.179 | 1.01 (0.99-1.02) | 0.351 |
| Age ≥65 years old | 1.94 (1.15-3.28) | **0.013** | 1.71 (1.00-2.95) | 0.051 | 1.57 (0.91-2.69) | 0.105 |
| Male | 1.10 (0.67-1.82) | 0.698 | 1.40 (0.85-2.29) | 0.184 | 1.00 (0.61-1.65) | 0.993 |
| BMI | 1.11 (1.04-1.19) | **0.001** | 1.14 (1.07-1.23) | **<0.001** | 1.12 (1.04-1.19) | **0.001** |
| Smoking | 0.91 (0.51-1.62) | 0.744 | 1.03 (0.58-1.82) | 0.919 | 1.03 (0.58-1.83) | 0.915 |
| **Underlying disease** |  |  |  |  |  |  |
| Diabetes mellitus | 1.41 (0.78-2.57) | 0.255 | 1.03 (0.57-1.89) | 0.912 | 1.57 (0.83-2.95) | 0.164 |
| Hypertension | 1.05 (0.62-1.78) | 0.861 | 1.07 (0.63-1.81) | 0.802 | 1.13 (0.66-1.92) | 0.653 |
| Human immunodeficiency virus | 1.63 (0.10-26.41) | 0.730 | 1087143447.7^#^ | 0.999 | 0.62 (0.04-10.05) | 0.738 |
| Lung disease | 0.59 (0.25-1.38) | 0.223 | 0.58 (0.27-1.27) | 0.173 | 0.64 (0.29-1.38) | 0.251 |
| Chronic obstructive pulmonary disease | 0.24 (0.05-1.07) | 0.060 | 0.42 (0.15-1.22) | 0.111 | 0.39 (0.14-1.14) | 0.086 |
| Asthma | 0.98 (0.23-4.17) | 0.974 | 0.66 (0.16-2.68) | 0.557 | 1.04 (0.24-4.45) | 0.956 |
| Old pulmonary tuberculosis | 0.9 (0.29-2.76) | 0.853 | 0.48 (0.16-1.42) | 0.185 | 0.61 (0.21-1.79) | 0.365 |
| Cirrhosis of liver | 2.01 (0.60-6.77) | 0.259 | 1.81 (0.47-6.99) | 0.388 | 1.10 (0.31-3.84) | 0.885 |
| End-stage renal disease | 0.98 (0.34-2.77) | 0.962 | 0.65 (0.24-1.78) | 0.399 | 0.46 (0.17-1.28) | 0.139 |
| Autoimmune disease | 0.84 (0.43-1.64) | 0.615 | 1.16 (0.60-2.24) | 0.652 | 1.21 (0.62-2.35) | 0.576 |
| **Cancer (hematologic and non-hematologic)** | 1.05 (0.60-1.85) | 0.854 | 1.00 (0.58-1.75) | 0.988 | 1.01 (0.58-1.76) | 0.978 |
| Hematologic malignancy | 0.79 (0.48-1.31) | 0.368 | 0.70 (0.42-1.16) | 0.162 | 0.63 (0.38-1.05) | 0.079 |
| without HSCT | 1.77 (1.04-3.01) | **0.034** | 1.80 (1.03-3.12) | **0.037** | 1.21 (0.70-2.07) | 0.493 |
| Non-hematologic malignancy | 1.53 (0.80-2.93) | 0.194 | 1.79 (0.89-3.60) | 0.101 | 2.50 (1.18-5.31) | 0.017 |
| **Organ transplant** | 0.29 (0.16-0.53) | **<0.001** | 0.29 (0.17-0.50) | **<0.001** | 0.44 (0.26-0.75) | **0.002** |
| HSCT | 0.29 (0.16-0.54) | **<0.001** | 0.32 (0.18-0.54) | **<0.001** | 0.42 (0.24-0.71) | **0.001** |
| Allogeneic HSCT | 0.31 (0.17-0.59) | **<0.001** | 0.35 (0.20-0.62) | **<0.001** | 0.48 (0.28-0.82) | **0.008** |
| Autologous HSCT | 0.26 (0.03-2.23) | 0.221 | 0.26 (0.05-1.35) | 0.108 | 0.24 (0.05-1.26) | 0.092 |
| Solid organ transplant | 0.32 (0.04-2.77) | 0.300 | 0.13 (0.01-1.11) | 0.062 | 0.62 (0.12-3.12) | 0.559 |
| Immunosuppressant or steroid use | 0.37 (0.22-0.62) | **<0.001** | 0.45 (0.28-0.75) | **0.002** | 0.52 (0.31-0.85) | **0.010** |
| Steroid | 0.64 (0.37-1.09) | 0.102 | 0.74 (0.44-1.24) | 0.257 | 0.72 (0.43-1.22) | 0.222 |
| Immunosuppressant | 0.29 (0.16-0.52) | **<0.001** | 0.38 (0.22-0.63) | **<0.001** | 0.55 (0.33-0.92) | **0.023** |
| Post Influenza | 1.42 (0.46-4.35) | 0.540 | 1.52 (0.46-5.08) | 0.492 | 1.43 (0.43-4.76) | 0.562 |
| White blood cell count, /μL | 1.00 (1.00-1.00) | 0.356 | 1.00 (1.00-1.00) | 0.805 | 1.00 (1.00-1.00) | 0.686 |
| Neutropenic status | 1.01 (0.62-1.66) | 0.962 | 0.95 (0.58-1.54) | 0.823 | 1.16 (0.70-1.90) | 0.567 |
| Proven IPA | 0.64 (0.19-2.09) | 0.456 | 0.35 (0.11-1.07) | 0.066 | 0.33 (0.11-1.00) | 0.051 |
| Positive *Aspergillus* culture | 1.76 (0.87-3.57) | 0.116 | 2.61 (1.14-5.96) | **0.023** | 2.43 (1.06-5.56) | **0.036** |
| *A.fumigatus* | 1.59 (0.71-3.53) | 0.258 | 2.03 (0.83-4.97) | 0.123 | 1.89 (0.77-4.65) | 0.164 |
| *A.flavus* | 1.65 (0.40-6.76) | 0.484 | 4.82 (0.58-39.74) | 0.144 | 4.52 (0.55-37.27) | 0.161 |
| *A.fumigatus* and *A.flavus* | 2655136870.43^#^ | 1.000^#^ | 1080348801.16^#^ | 1.000^#^ | 1014597004.96^#^ | 1.000^#^ |
| **Serum Galactomannan assay** |  |  |  |  |  |  |
| Baseline sGMI | 1.19 (1.05-1.35) | **0.005** | 1.27 (1.11-1.46) | **0.001** | 1.26 (1.10-1.45) | **0.001** |
| Baseline sGMI ≥1.5 | 1.66 (1.01-2.74) | **0.045** | 2.2 (1.34-3.63) | **0.002** | 2.23 (1.35-3.69) | **0.002** |
| Baseline sGMI ≥2 | 1.94 (1.17-3.20) | **0.010** | 2.2 (1.31-3.69) | **0.003** | 2.46 (1.45-4.17) | **0.001** |
| Day 7 sGMI ≥1.5 | 2.56 (1.33-4.94) | **0.005** | 2.69 (1.42-5.09) | **0.002** | 2.44 (1.29-4.60) | **0.006** |
| Day 7 sGMI- baseline sGMI | 1.14 (0.97-1.32) | 0.105 | 1.15 (0.99-1.34) | 0.073 | 1.08 (0.93-1.25) | 0.324 |
| Day 14 sGMI- baseline sGMI | 1.21 (1.00-1.46) | 0.053 | 1.13 (0.96-1.33) | 0.156 | 1.04 (0.89-1.23) | 0.614 |
| Maximum sGMI- baseline sGMI | 1.15 (0.94-1.40) | 0.172 | 1.27 (1.01-1.60) | **0.037** | 1.18 (0.95-1.46) | 0.139 |
| Week 2 – Week 1 sGMI | 1.22 (0.91-1.64) | 0.186 | 0.92 (0.71-1.18) | 0.506 | 0.89 (0.69-1.15) | 0.379 |
| Doubling in sGMI | 1.19 (0.58-2.43) | 0.629 | 1.52 (0.74-3.14) | 0.253 | 1.65 (0.79-3.44) | 0.180 |
| sGMI persistently ≥1.0 | 4.07 (2.06-8.05) | **<0.001** | 2.48 (1.35-4.53) | **0.003** | 1.54 (0.85-2.80) | 0.155 |
| Increasing trajectory in sGMI within 28 days | 3.02 (1.6-5.72) | **0.001** | 2.11 (1.1-4.02) | **0.024** | 1.59 (0.84-3) | 0.153 |
| **Antifungal treatment** | 0.34 (0.16-0.74) | **0.006** | 0.58 (0.26-1.31) | 0.192 | 0.62 (0.27-1.41) | 0.256 |
| Voriconazole | 0.46 (0.27-0.79) | **0.005** | 0.79 (0.46-1.36) | 0.389 | 0.68 (0.39-1.18) | 0.170 |
| Other treatment | 1.40 (0.74-2.64) | 0.305 | 0.98 (0.51-1.85) | 0.939 | 1.26 (0.65-2.44) | 0.496 |
| **Radiographic finding** |  |  |  |  |  |  |
| Consolidation | 2.22 (1.26-3.92) | **0.006** | 2.36 (1.39-3.99) | **0.001** | 2.61 (1.54-4.43) | **<0.001** |
| Nodules | 0.90 (0.53-1.51) | 0.686 | 1.06 (0.63-1.79) | 0.818 | 0.94 (0.56-1.59) | 0.826 |
| Halo sign | 0.90 (0.54-1.51) | 0.687 | 0.84 (0.50-1.39) | 0.487 | 0.87 (0.52-1.44) | 0.582 |
| Mass | 1.68 (0.95-2.97) | 0.075 | 1.21 (0.67-2.16) | 0.527 | 0.93 (0.52-1.66) | 0.816 |
| Cavitation | 1.28 (0.63-2.58) | 0.497 | 1.25 (0.61-2.59) | 0.541 | 1.02 (0.50-2.08) | 0.961 |
| ICU Admission | 1.79 (1.04-3.08) | **0.037** | 2.07 (1.23-3.47) | **0.006** | 2.84 (1.68-4.80) | **<0.001** |

Abbreviations: OR= Odds ratio; 95% CI= 95% confidence interval; BMI= body mass index; HSCT= hematopoietic stem cell transplantation; IPA=invasive pulmonary aspergillosis; sGMI= serum galactomannan enzyme immunoassay optical density index

***p* value <0.05**= Bold underlined

^#^ Perfect separation of data with all subjects fall within mortality group, unable to analysis

**Supplementary Figure 3.** Receiver operating characteristic curve of the sGMI markers that were significant in multivariable logistic regression


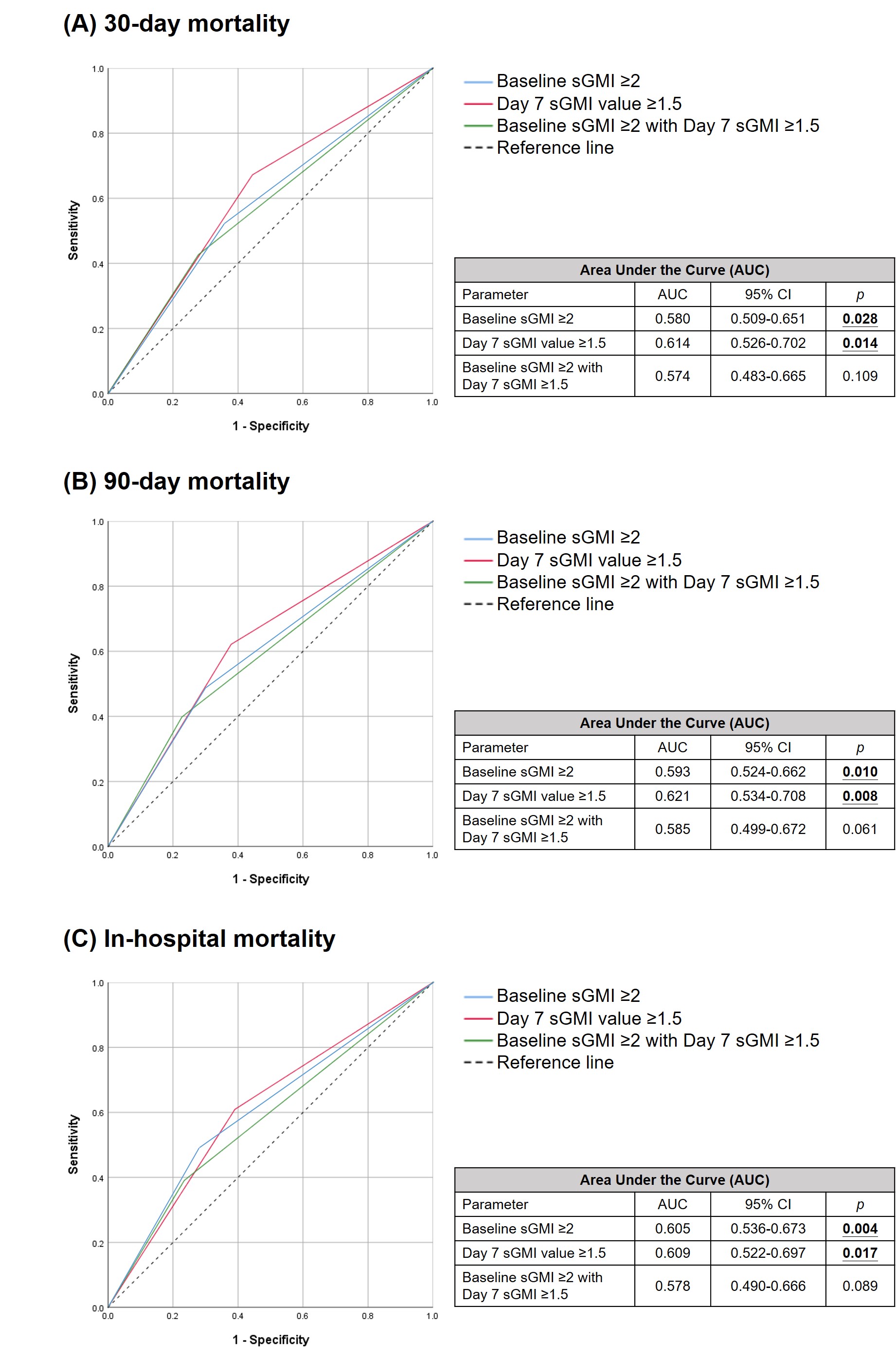


Abbreviation: sGMI= serum galactomannan enzyme immunoassay optical density index

***p* value <0.05**= Bold underlined

**Supplementary Figure 4.** Receiver operating characteristic curve of the multivariable logistic regression model performance with significant sGMI

**
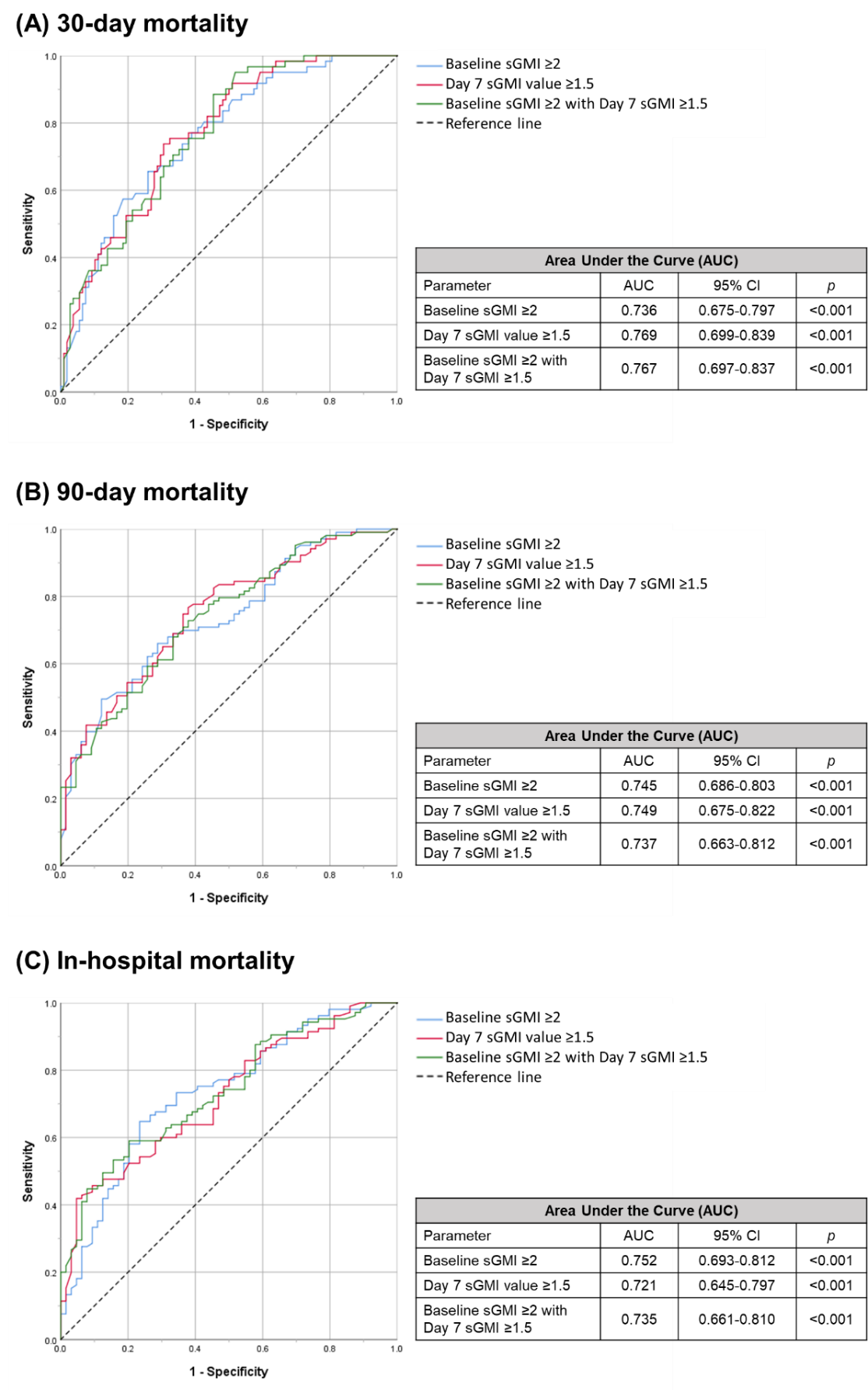
**

Abbreviation: sGMI= serum galactomannan enzyme immunoassay optical density index

**Supplementary Information 1:** Logistic regression model development, specification details

Assuming a power of 0.8, with half of the patients harboring the predictive marker and a 60% mortality rate. With an odds ratio of 3, the estimated sample size for the total cohort was 88 patients. Our cohort has surpassed the estimated number of required participants with 268 patients included in analysis.

We adhered to the rule of having at least ten events per regression coefficient estimated. The changes in the Akaike’s information criterion (AIC) and Bayesian information criterion (BIC) was considered during the process. Different static and kinetic GMI markers were implemented into the final model one at a time to check if the marker is statistically significant for outcome prediction. We examined Spearman correlations and variance inflation factors among the variables in the model to avoid collinearity. There were missing sGMI data at subsequent follow-up due to mortality before designed endpoint but no imputation or deletion were done due to the nature of our study design.

The full prediction model of baseline sGMI ≥2 and Day 7 sGMI ≥1.5 with regression coefficient, model instant, Hosmer-Lemeshow test, AIC, and BIC are reported in the following tables **Supplementary Information 1 Table A**.

**Supplementary Information 1 Table A:** Full multivariable logistic regression model with baseline sGMI ≥2 and day 7 sGMI ≥1.5 as marker

| **Baseline sGMI ≥2** | 30-day mortality | | | 90-day mortality | | | In-hospital mortality | | | |
| --- | --- | --- | --- | --- | --- | --- | --- | --- | --- | --- |
|  | β | OR (95% CI) | *p* | β | OR (95% CI) | *p* | β | OR (95% CI) | | *p* |
| **Baseline sGMI ≥2** (n=268) | 0.725 | 2.06 (1.16-3.66) | **0.013** | 0.846 | 2.33 (1.29-4.21) | **0.005** | 1.095 | 2.99 (1.62-5.51) | | **<0.001** |
| Age | -0.002 | 1.00 (0.98-1.02) | 0.793 | -0.013 | 0.99 (0.97-1.01) | 0.170 | -0.014 | 0.99 (0.97-1.00) | | 0.122 |
| Male (n=151) | -0.118 | 0.89 (0.51-1.56) | 0.681 | 0.194 | 1.21 (0.69-2.14) | 0.503 | -0.28 | 0.76 (0.42-1.35) | | 0.344 |
| BMI | 0.071 | 1.07 (1.00-1.15) | 0.050 | 0.1 | 1.11 (1.02-1.19) | **0.010** | 0.078 | 1.08 (1.00-1.17) | | **0.046** |
| HSCT (n=81) | -0.098 | 0.46 (0.20-1.07) | 0.071 | 0.138 | 0.39 (0.17-0.88) | **0.024** | 0.672 | 0.54 (0.23-1.26) | | 0.153 |
| Solid organ transplant (n=6) | 0.009 | 0.28 (0.03-2.83) | 0.284 | 0.181 | 0.07 (0.01-0.66) | 0.021 | -0.217 | 0.40 (0.07-2.49) | | 0.328 |
| Hematologic malignancy without HSCT (n=82) | -0.78 | 1.01 (0.45-2.26) | 0.982 | -0.944 | 1.20 (0.51-2.80) | 0.675 | -0.612 | 0.81 (0.34-1.91) | | 0.624 |
| Non-hematologic malignancy (n=45) | -1.258 | 0.91 (0.41-1.99) | 0.808 | -2.722 | 1.15 (0.48-2.73) | 0.755 | -0.909 | 1.96 (0.78-4.92) | | 0.153 |
| Immunosuppressant or steroid use (n=124) | -0.616 | 0.54 (0.28-1.05) | 0.069 | -0.191 | 0.83 (0.43-1.60) | 0.571 | -0.357 | 0.70 (0.35-1.39) | | 0.307 |
| Neutropenic status(n=123) | 0.201 | 1.22 (0.66-2.27) | 0.523 | 0.131 | 1.14 (0.61-2.12) | 0.677 | 0.64 | 1.90 (1.00-3.60) | | 0.050 |
| Consolidation pattern on CT (n=183) | 0.57 | 1.77 (0.91-3.44) | 0.093 | 0.617 | 1.85 (0.98-3.50) | 0.057 | 0.759 | 2.14 (1.13-4.05) | | **0.020** |
| Anti-fungal treatment (n=237) | -0.956 | 0.38 (0.17-0.88) | **0.025** | -0.228 | 0.80 (0.32-1.97) | 0.621 | -0.2 | 0.82 (0.33-2.06) | | 0.67 |
| ICU admission (n=179) | 0.46 | 1.58 (0.84-2.98) | 0.153 | 0.753 | 2.12 (1.15-3.92) | **0.016** | 1.167 | 3.21 (1.71-6.02) | | **<0.001** |
| Constant | -1.659 |  | 0.157 | -1.913 |  | 0.109 | -1.726 |  | | 0.155 |
| HL goodness of fit, Chi-square (df) | 1.876 (8) |  | 0.985 | 7.857 (8) |  | 0.448 | 1.757 (8) |  | | 0.988 |
| AIC | 340.11 |  |  | 335.15 |  |  | 332.56 |  | |  |
| BIC | 390.37 |  |  | 385.42 |  |  | 382.83 |  | |  |
|  |  | | |  | | |  | | | |
| **Day 7 sGMI ≥1.5** | 30-day mortality | | | 90-day mortality | | | In-hospital mortality | | | |
|  | β | OR (95% CI) | *p* | β | OR (95% CI) | *p* | β | OR (95% CI) | *p* | |
| **Day 7 sGMI ≥1.5** (n=169) | 0.851 | 2.34 (1.09-5.02) | **0.029** | 0.807 | 2.24 (1.10-4.58) | **0.027** | 0.832 | 2.30 (1.12-4.71) | **0.023** | |
| Age | -0.008 | 0.99 (0.97-1.02) | 0.543 | -0.014 | 0.99 (0.96-1.01) | 0.276 | -0.016 | 0.98 (0.96-1.01) | 0.208 | |
| Sex (n=93) | -0.446 | 0.64 (0.30-1.36) | 0.244 | -0.106 | 0.90 (0.43-1.86) | 0.776 | -0.57 | 0.57 (0.27-1.19) | 0.131 | |
| BMI | 0.075 | 1.08 (0.98-1.18) | 0.113 | 0.156 | 1.17 (1.06-1.29) | **0.002** | 0.123 | 1.13 (1.03-1.25) | **0.013** | |
| HSCT (n=50) | 0.015 | 0.77 (0.23-2.53) | 0.666 | 0.092 | 0.69 (0.24-2.02) | 0.499 | 0.676 | 1.17 (0.39-3.50) | 0.777 | |
| Solid organ transplant (n=5) | 0.412 | 0.75 (0.06-9.69) | 0.827 | 0.251 | 0.19 (0.02-2.09) | 0.175 | -0.099 | 1.60 (0.22-11.78) | 0.647 | |
| Hematologic malignancy without HSCT (n=47) | -0.263 | 1.51 (0.50-4.54) | 0.463 | -0.370 | 1.29 (0.41-4.01) | 0.665 | 0.158 | 0.91 (0.29-2.85) | 0.866 | |
| Non-hematologic malignancy (n=32) | -0.285 | 1.02 (0.37-2.82) | 0.977 | -1.650 | 1.10 (0.38-3.17) | 0.865 | 0.467 | 1.97 (0.66-5.87) | 0.225 | |
| Immunosuppressant or steroid use (n=80) | -1.048 | 0.35 (0.14-0.90) | **0.029** | -0.496 | 0.61 (0.25-1.50) | 0.279 | -0.660 | 0.52 (0.21-1.30) | 0.161 | |
| Neutropenic status (n=75) | 0.075 | 1.08 (0.47-2.48) | 0.859 | -0.086 | 0.92 (0.41-2.04) | 0.834 | 0.509 | 1.66 (0.74-3.74) | 0.218 | |
| Consolidation pattern on CT (n=120) | 1.065 | 2.90 (1.10-7.64) | **0.031** | 0.525 | 1.69 (0.74-3.89) | 0.217 | 0.852 | 2.35 (1.02-5.38) | **0.044** | |
| Anti-fungal treatment (n=154) | -0.994 | 0.37 (0.11-1.28) | 0.117 | 0.420 | 1.52 (0.43-5.34) | 0.512 | 0.244 | 1.28 (0.36-4.54) | 0.707 | |
| ICU admission (n=116) | 0.920 | 2.51 (0.99-6.34) | 0.052 | 0.740 | 2.10 (0.93-4.71) | 0.073 | 0.990 | 2.69 (1.19-6.11) | **0.018** | |
| Constant | -2.244 |  | 0.177 | -3.409 |  | 0.034 | -2.928 |  | 0.071 | |
| HL goodness of fit, Chi-square (df) | 6.622 (8) |  | 0.578 | 7.891 (8) |  | 0.444 | 7.304 (8) |  | 0.504 | |
| AIC | 210.13 |  |  | 220.47 |  |  | 224.56 |  |  | |
| BIC | 253.95 |  |  | 264.29 |  |  | 268.38 |  |  | |

**Abbreviations:** β= beta coefficient; aOR= adjusted odds ratio; 95% CI= 95% confidence interval; HSCT= hematopoietic stem cell transplantation; sGMI= serum galactomannan enzyme immunoassay optical density index; ICU= Intensive care unit; HL=Hosmer-Lemeshow test; df= degree of freedom; AIC= Akaike’s information criterion; BIC=Bayesian information criterion

*p* value <0.05: **Bold underline**

**Supplementary Information 2:** References of the Systematic Review of static and kinetic serum Galactomannan markers

1. Heylen L, Maertens J, Naesens M, Van Wijngaerden E, Lagrou K, Bammens B, et al. Invasive aspergillosis after kidney transplant: case-control study. Clin Infect Dis. 2015;60(10):1505-11.

DOI: https://doi.org/10.1093/cid/civ103

2. Koo S, Bryar JM, Baden LR, Marty FM. Prognostic features of galactomannan antigenemia in galactomannan-positive invasive aspergillosis. J Clin Microbiol. 2010;48(4):1255-60.

DOI: https://doi.org/10.1128/JCM.02281-09

3. Bergeron A, Porcher R, Menotti J, Poirot JL, Chagnon K, Vekhoff A, et al. Prospective evaluation of clinical and biological markers to predict the outcome of invasive pulmonary aspergillosis in haematological patients. J Clin Microbiol. 2012;50(3):823-30.

DOI: https://doi.org/10.1128/JCM.00750-11

4. Pang L, Zhao X, Dickens BL, Lim JT, Cook AR, Netea MG, et al. Using routine blood parameters to anticipate clinical outcomes in invasive aspergillosis. Clin Microbiol Infect. 2020;26(6):781 e1- e8.

DOI: https://doi.org/10.1016/j.cmi.2019.10.019

5. Couchepin J, Brunel AS, Jaton K, Meylan P, Bochud PY, Lamoth F. Role of bi-weekly serum galactomannan screening for the diagnosis of invasive aspergillosis in haaematological cancer patients. Mycoses. 2018;61(6):350-4.

DOI: https://doi.org/10.1111/myc.12755

6. Neofytos D, Railkar R, Mullane KM, Fredricks DN, Granwehr B, Marr KA, et al. Correlation between Circulating Fungal Biomarkers and Clinical Outcome in Invasive Aspergillosis. PLoS One. 2015;10(6):e0129022.

DOI: https://doi.org/10.1371/journal.pone.0129022

7. Hoyo I, Sanclemente G, de la Bellacasa JP, Cofan F, Ricart MJ, Cardona M, et al. Epidemiology, clinical characteristics, and outcome of invasive aspergillosis in renal transplant patients. Transpl Infect Dis. 2014;16(6):951-7.

DOI: https://doi.org/10.1111/tid.12301

8. Moreno A, Mah J, Budvytiene I, Ho Dora Y, Schwenk Hayden T, Banaei N. Dynamics and prognostic value of plasma cell-free DNA PCR in patients with invasive aspergillosis and mucormycosis. Journal of Clinical Microbiology. 2024;62(5):e00394-24.

DOI: https://doi.org/10.1128/jcm.00394-24

9. Fisher CE, Stevens AM, Leisenring W, Pergam SA, Boeckh M, Hohl TM. The serum galactomannan index predicts mortality in haematopoietic stem cell transplant recipients with invasive aspergillosis. Clin Infect Dis. 2013;57(7):1001-4.

DOI: https://doi.org/10.1093/cid/cit393

10. Kim SJ, Cheong JW, Min YH, Choi YJ, Lee DG, Lee JH, et al. Success rate and risk factors for failure of empirical antifungal therapy with itraconazole in patients with haematological malignancies: a multicenter, prospective, open-label, observational study in Korea. J Korean Med Sci. 2014;29(1):61-8.

DOI: https://doi.org/10.3346/jkms.2014.29.1.61

11. Han SB, Kim SK, Lee JW, Yoon JS, Chung NG, Cho B, et al. Serum galactomannan index for early prediction of mortality in immunocompromised children with invasive pulmonary aspergillosis. BMC infectious diseases. 2015;15:271.

DOI: https://doi.org/10.1186/s12879-015-1014-9

12. Vehreschild JJ, Heussel CP, Groll AH, Vehreschild MJGT, Silling G, Würthwein G, et al. Serial assessment of pulmonary lesion volume by computed tomography allows survival prediction in invasive pulmonary aspergillosis. European Radiology. 2017;27(8):3275-82.

DOI: https://doi.org/10.1007/s00330-016-4717-4

13. Lopez-Medrano F, Fernandez-Ruiz M, Silva JT, Carver PL, van Delden C, Merino E, et al. Clinical Presentation and Determinants of Mortality of Invasive Pulmonary Aspergillosis in Kidney Transplant Recipients: A Multinational Cohort Study. Am J Transplant. 2016;16(11):3220-34.

DOI: https://doi.org/10.1111/ajt.13837

14. Chai LY, Kullberg BJ, Johnson EM, Teerenstra S, Khin LW, Vonk AG, et al. Early serum galactomannan trend as a predictor of outcome of invasive aspergillosis. J Clin Microbiol. 2012;50(7):2330-6.

DOI: https://doi.org/10.1128/JCM.06513-11

15. Nouer SA, Nucci M, Kumar NS, Grazziutti M, Restrepo A, Anaissie E. Baseline platelet count and creatinine clearance rate predict the outcome of neutropenia-related invasive aspergillosis. Clin Infect Dis. 2012;54(12):e173-83.

DOI: https://doi.org/10.1093/cid/cis298

16. Chai LY, Kullberg BJ, Earnest A, Johnson EM, Teerenstra S, Vonk AG, et al. Voriconazole or amphotericin B as primary therapy yields distinct early serum galactomannan trends related to outcomes in invasive aspergillosis. PLoS One. 2014;9(2):e90176.

DOI: https://doi.org/10.1371/journal.pone.0090176

17. Park SY, Lim C, Lee SO, Choi SH, Kim YS, Woo JH, et al. Computed tomography findings in invasive pulmonary aspergillosis in non-neutropenic transplant recipients and neutropenic patients, and their prognostic value. J Infect. 2011;63(6):447-56.

DOI: https://doi.org/10.1016/j.jinf.2011.08.007

18. Woods G, Miceli MH, Grazziutti ML, Zhao W, Barlogie B, Anaissie E. Serum Aspergillus galactomannan antigen values strongly correlate with outcome of invasive aspergillosis: a study of 56 patients with haematologic cancer. Cancer. 2007;110(4):830-4.

DOI: https://doi.org/10.1002/cncr.22863

19. Maertens J, Buve K, Theunissen K, Meersseman W, Verbeken E, Verhoef G, et al. Galactomannan serves as a surrogate endpoint for outcome of pulmonary invasive aspergillosis in neutropenic haematology patients. Cancer. 2009;115(2):355-62.

DOI: https://doi.org/10.1002/cncr.24022

20. Nouer SA, Nucci M, Kumar NS, Grazziutti M, Barlogie B, Anaissie E. Earlier response assessment in invasive aspergillosis based on the kinetics of serum Aspergillus galactomannan: proposal for a new definition. Clin Infect Dis. 2011;53(7):671-6.

DOI: https://doi.org/10.1093/cid/cir441

21. Park SH, Choi SM, Lee DG, Choi JH, Kim SH, Kwon JC, et al. Serum galactomannan strongly correlates with outcome of invasive aspergillosis in acute leukaemia patients. Mycoses. 2011;54(6):523-30.

DOI: https://doi.org/10.1111/j.1439-0507.2010.02009.x

22. Salonen J, Lehtonen OP, Terasjarvi MR, Nikoskelainen J. Aspergillus antigen in serum, urine and bronchoalveolar lavage specimens of neutropenic patients in relation to clinical outcome. Scandinavian journal of infectious diseases. 2000;32(5):485-90.

DOI: https://doi.org/10.1080/003655400458749

23. Chen WC, Chen IC, Chen JP, Liao TL, Chen YM. Prognostic factors and outcomes of invasive pulmonary aspergillosis, a retrospective hospital-based study. PeerJ. 2024;12:e17066.

DOI: https://doi.org/10.7717/peerj.17066

24. Teering S, Verreth A, Peeters A, Van Regenmortel N, De Laet I, Schoonheydt K, et al. Prognostic value of serum galactomannan in mixed ICU patients: a retrospective observational study. Anaesthesiology intensive therapy. 2014;46(3):145-54.

DOI: https://doi.org/10.5603/ait.2014.0027

25. Khanna S, Oberoi JK, Datta S, Aggarwal S, Wattal C. Variables affecting the performance of galactomannan assay in high-risk patients at a tertiary care centre in India. Indian J Med Microbiol. 2013;31(1):34-9.

DOI: https://doi.org/10.4103/0255-0857.108717

26. Dabas Y, Mohan A, Xess I. Serum galactomannan antigen as a prognostic and diagnostic marker for invasive aspergillosis in heterogeneous medicine ICU patient population. PLoS One. 2018;13(4):e0196196.

DOI: https://doi.org/10.1371/journal.pone.0196196

27. Mercier T, Wera J, Chai LYA, Lagrou K, Maertens J. A Mortality Prediction Rule for Haematology Patients with Invasive Aspergillosis Based on Serum Galactomannan Kinetics. J Clin Med. 2020;9(2).

DOI: https://doi.org/10.3390/jcm9020610

28. Er B, Er AG, Gulmez D, Sahin TK, Metan G, Saribas Z, et al. Diagnostic performance and longitudinal analysis of fungal biomarkers in COVID-19 associated pulmonary aspergillosis. Heliyon. 2023;9(11):e21721.

DOI: https://doi.org/10.1016/j.heliyon.2023.e21721
